# Omicron: the highly mutational COVID-19 variant with immune escape

**DOI:** 10.11604/pamj.2022.41.84.33373

**Published:** 2022-01-31

**Authors:** Rumi Khajotia

**Affiliations:** 1 Internal Medicine, International Medical University, Seremban, Malaysia

**Keywords:** Omicron, COVID-19, COVID-19 variant

## Abstract

In the latter-half of 2021, as people all over the world began optimistically thinking that reopening was just a heartbeat away, providence meant otherwise, and the world was once again hit by a COVID-19 variant; this time with a record number of 32 mutations across its spike proteins and significantly increased transmissibility, infectiousness and immune escape. The WHO subsequently named this variant the “Omicron variant,” after yet another new Greek alphabet. Subsequently, it has been observed that the reinfection (evasion of immunity derived from prior infection) risk from the Omicron variant of the SARS-CoV-2 virus is substantially higher than from the previously identified beta and delta variants. South African researchers have found preliminary results suggesting significant and ongoing increase in the risk of reinfection with the Omicron variant in patients who previously suffered from COVID-19 infection.

Just as the world was eagerly looking forward to a much-anticipated global reopening, providential dispensation proved otherwise. A new variant with increased transmissibility emerged, especially among those with prior COVID-19 infection, indicating that here was a SARS-CoV-2 variant which had the ability to escape immunity with impunity! Though this new variant B.1.1.529 was first discovered on 24^th^ November 2021, presumably in an immunocompromised patient in South Africa, the first confirmed infection with B.1.529 was detected in a specimen dating back to November 09, 2021. However, researchers now believe it could have been circulating in the global population for a much longer period of time, since there is evidence emerging of community transmission among people with no history of travel to South Africa or any other African country. It was also discovered that this new B.1.1.529 variant was unlike any before it, in that it had a record number of more than 50 mutations, including more than 30 mutations on its spike proteins, which is the vehicle that the virus uses to attach to human cells and thereby gain entry to the body and cause illness. Since the spike protein is the main target of the antibodies which are produced by the immune system to fight a COVID-19 infection, such a plethora of mutations raised concerns among scientists that Omicron´s spike proteins may be able to evade antibodies produced by patients as a direct result of prior infection or vaccination.

Consequently, the WHO immediately swung into action and on 26^th^ November 2021 designated this highly mutational SARS-CoV-2 variant as a “variant of concern” (VOC) [1] and named it after yet another Greek alphabet “*Omicron*,” on the advice of its Technical Advisory Group on Virus Evolution (TAG-VE) [2].

As of 1^st^ December 2021, at least 23 countries had reported cases involving the Omicron variant of SARS-CoV-2. The U.S. was the 24^th^ country to report its first cases of the Omicron variant in a fully vaccinated man in California. This was confirmed by the Centers for Disease Control and Prevention. A second case of the Omicron variant emerged in the US in a man from Minnesota who had recently travelled to New York to attend a convention. He too was fully vaccinated and is not in any serious condition at present.

Other countries which have reported cases of the Omicron variant include the UK, Canada, France, Sweden, Australia, Denmark, Germany, Holland, Italy, India, Belgium and Malaysia. Malaysia detected its first case in a man who came from Singapore and had a history of recent travel to South Africa. However, since there is now overwhelming evidence of community transmission of the Omicron variant, with cases emerging among people with no history of travel to the African continent or contact with a person with a recent history of travel to South Africa, researchers believe that the Omicron variant of SARS-CoV-2 has possibly been circulating undetected among the global population for some significant period of time. Consequently, on 2^nd^ December 2021, the WHO´s Africa Office declared that the exact origins of the Omicron variant are essentially unknown as the “surveillance system in the global world is not perfect yet.” They mentioned that South Africa first detected the Omicron variant possibly because it has a good surveillance system in place with a high rate of genome sequencing done on people with symptoms.

Dr. Moritz Kraemer, lead researcher on the Oxford Martin Programme on Pandemic Genomics at Oxford University agreed that early detection of the Omicron variant in South Africa was possibly because South Africa has a robust genomic sequencing and surveillance programme in place, unlike many other nations. Hence, it is possible that the Omicron variant could have originated much earlier elsewhere and was only now first detected in South Africa. Dr. Angelique Coetzee who is the chairperson of the South African Medical Association was the doctor who first suspected the possibility of a new COVID-19 variant in the Gauteng province in South Africa, when she first saw patients on November 18, 2021 with symptoms that differed from those seen in patients infected with other COVID-19 variants. She first saw these symptoms in a 33-year-old man who complained of nothing more than severe generalised body ache, extreme tiredness and a mild headache. The patient only had a 'scratchy throat' with no cough or loss of sense of smell or taste. Test for COVID-19 was positive in this patient. Consequently, Dr. Coetzee saw more cases with similar atypically 'mild symptoms' that day, which were different from symptoms in COVID-19 patients. This caused her to alert the South African Vaccine Advisory Committee leading to genomic sequencing of the virus, and the consequent discovery of a new variant of the SARS-CoV-2, namely, the Omicron variant.

In the coming days, Dr. Coetzee and her colleagues saw more such cases with 'mild symptoms' who did not require any hospitalisation. However, Dr. Coetzee's observations are only based on a small number of cases and virologists worry that given Omicron's large number of spike protein mutations, the symptomatology and severity of infection could dramatically change in the coming weeks and months, as was earlier also the case with the beta and delta variants of the COVID-19 virus. Genomic sequencing of these early cases in South Africa indicates that the Omicron variant of SARS-CoV-2 has an S-gene target failure or S-gene dropout on PCR assay due to a 69-70 del deletion [3]. The PCR test can therefore be used as a marker for the Omicron variant while we are still awaiting full sequencing confirmation.

It is believed that the Omicron variant has increased rate of transmission, greater viral binding and a higher antibody escape [4,5] due to multiple deletions and more than 30 mutations on its spike proteins such as, 69-70 del, N501Y, N679K, G142D/143-145del, K417N, P681H, T478K and T951, some of which also overlap with mutations in the alpha, beta, gamma and delta variants of SARS-CoV-2 [6]. However, the deleterious effects of some of the other mutations and deletions which are specific only to the Omicron variant are as yet unknown, resulting in a high level of uncertainty as to what will be their implication on viral behaviour, immune escape, and vaccine-mediated immunity [4,5].

If the Omicron mutations maintain their known effects, increased transmissibility remains a concern, especially because of mutations near the furin cleavage site. Since the past 2 weeks, epidemiological evidence coming out of South Africa suggests a sudden surge in COVID-19 cases, along with a rising rate of PCR tests showing S-gene target failure. However, it is also known that when viruses accumulate a lot of mutations they may lose some of their ability to cleave, which in turn may change the behaviour of their spike proteins. Some scientists are therefore theorising that Omicron may have mutated over many months in an immunocompromised individual, such as an HIV patient, and thereby 'adapted not to kill the host,' according to Dr. John Wherry, director of the Penn Institute of Virology in Philadelphia. This may be one of the reasons why most patients seem to be suffering 'mild symptoms' when infected with the Omicron variant, requiring little or no hospitalisation.

It is also being hypothesized by researchers that if the Omicron variant has a predilection towards reinfecting patients who already have had COVID-19 in the past, it is possible that the disease could run a mild course without significant morbidity and mortality, as these patients would already be having a fair degree of immunity due to past infection. However, immune escape remains a major concern with the Omicron variant due to its predilection to cause reinfection in patients with a past history of COVID-19 infection. This is in keeping with the immune-escape mutations already observed in the Omicron variant. Previous studies have shown that although most deletions and mutations in the SARS-CoV-2 genome are either deleterious but quickly neutralized, or relatively harmless, a small number of these mutations and deletions significantly affect viral functions, thereby increasing infectivity, transmissibility, severity of illness and interactions with the host immunity.

As yet, it is not fully known to what extent mutations and deletions affecting the antigenic phenotype of SARS-CoV-2 will enable variants such as Omicron to escape immunity got either by a natural infection or following vaccination. However, recent research has shown that deletions and mutations that change the antigenic phenotype of the COVID-19 virus are now present worldwide and are significantly affecting immune recognition to an alarming degree. As is evident, the spike protein facilitates attachment of the virus to the host cell- surface receptors resulting in fusion between the virus and host cell membranes [7]. The spike protein is also the principal target of neutralizing antibodies which develop following infection by the SARS-CoV-2 virus [8,9] and is the SARS-CoV-2 component of both mRNA and adenovirus-based vaccines which are now in use [10]. Hence it is of concern that mutations affecting the antigenicity of the spike protein could reduce the efficacy of the existing vaccines.

However, until now, for all preceding variants, most COVID-19 vaccines have remained effective in preventing severe COVID-19 infection. This may be because this efficacy is more dependent on T-cell immune responses than antibodies. Previous studies conducted in Qatar and the US [11,12] have reported vaccine efficacy of > 90% in preventing severe infections requiring hospitalization during the surge in cases due to the delta-variant. This was also seen up to 6 months after full vaccination which was encouraging.

Knowledge of spike protein mutations is rooted in the study of the amino acid changes which are increasing in frequency and are characterised by unusual epidemiological features. A noteworthy mutation in spike proteins was N439K as it enhanced the binding affinity for the ACE2 receptor and reduced the neutralizing activity of some monoclonal antibodies (mAbs) and polyclonal antibodies present in the serum of patients who had recovered following a COVID-19 infection. Another receptor-binding motif (RBM) amino acid change, Y453F, which was associated with increased binding for ACE2 receptors received significant attention after it was identified in sequences associated with increased infections in Denmark and initially named ‘cluster 5´ (now B.1.1.298) [13].

Scientists believe genomic sequencing has shown a change in host environment and evidence of increased selective pressures acting upon immunologically significant COVID-19 genes which were sampled over the course of many months. This has coincided with the emergence of variants with increasing numbers of mutations. Because of their association with increased transmissibility, these lineages were named “variants of concern” (VOC). The variants of concern are identified by multiple convergent mutations that are believed to have occurred either because of chronic infections or in patients previously infected by the COVID-19 virus [14].

The entry of SARS-CoV-2 into host cells is facilitated by the transmembrane spike glycoprotein, which forms homotrimers on the surface of the virion. This spike protein is highly glycosylated. It has 66 potential N-glycosylation sites per trimer [15]. In addition, the spike protein is post-translationally cleaved by mammalian furin into two subunits: S1 and S2. The S1 subunit predominantly consists of the amino-terminal domain and the receptor-binding domain (RBD). The S1 subunit is primarily responsible for binding to the host cell-surface receptor, ACE2. In contrast, the S2 subunit includes the trimeric core of the protein and is responsible for membrane fusion. It is the presence of a polybasic furring cleavage site at the S1-S2 boundary which is responsible for the infectivity and virulence of the COVID-19 virus [16].

Consequently, several studies have probed the antigenicity of the SARS-CoV-2 spike protein by a process known as epitope mapping. In one study, serological analyses of 650 individuals infected with SARS-CoV-2 indicated that nearly 90% of the serum neutralizing antibody activity targeted the spike receptor-binding domain (RBD) [8]. With much research material available regarding spike proteins, researchers are now attempting to measure Omicron´s ability to evade immune responses. One team of virologists at the NICD and the University of Witwatersrand in Johannesburg is measuring the ability of virus-blocking (or neutralizing) antibodies in preventing Omicron from infected fresh human host cells.

In one significant study in Nature [17], a team of scientists engineered a highly mutated version of the spike protein in a virus incapable of causing COVID-19, that shares many of the mutations with Omicron. This “polymutant spike” proved to be completely resistant to neutralizing antibodies derived from majority of the people who had either recovered from COVID-19 or had received two doses of an mRNA vaccine. These results would not bode well for our global fight against the SARS-CoV-2, if the Omicron variant were to develop “polymutant spikes,” as a result of further mutations and deletions on its spike proteins.

Latest information suggests that a new version of the Omicron coronavirus variant has been found which scientists believe will be harder to detect because of its genetic makeup. The new lineage now labelled BA.2, has been discovered in South Africa, Canada and Australia [18]. BA.2 is genetically quite different from the original Omicron lineage known as BA.1 in that it does not possess the S-gene dropout mutation which has facilitated Omicron BA.1 variant to be detected by PCR testing. This indicates that the behaviour patterns of the two lineages of the Omicron coronavirus variant may be different. BA.2 is believed to be carrying many of the characteristic mutations which define the Omicron variant; however, it also has many more mutations which BA.1 does not possess and has also dropped many of the mutations which appear on BA.1.

A main characteristic of BA.2 is that it is lacking the 69/70del mutation on the S-gene, which is the defining feature of the BA.1 Omicron variant [18]. Normally, PCR tests check for different markers one of which targets the S-gene. Hence, in a patient with the BA.1 Omicron lineage one of the markers will not function; this is called an S-gene dropout. This helps to differentiate Omicron from other coronavirus variants, most of which do not cause an S-gene dropout. However, due to the absence of the S-gene dropout in the BA.2 lineage of the Omicron variant detection will in this case have to depend on genomic sequencing to identify it. This may also indicate that there may already be more Omicron circulating among the global population than we believe to be the case. Despite this, PCR tests will continue to help in detecting the coronavirus per se, even with this new lineage.

**Latest updates:** latest statistics from the US indicate that the highly contagious Omicron variant of SARS-CoV-2 is now responsible for the majority of cases in the country. Also, the seven-day rolling average for daily new COVID-19 deaths has now reached 2267 as of Thursday, September 27, 2022 [19]. Although patients infected by the Omicron variant of SARS-CoV-2 usually show mild symptoms or no symptoms at all, it is now observed that Omicron can be deadly in patients who are unvaccinated, are older and who have comorbidities. Meanwhile, the WHO has said that there were 21 million new COVID-19 cases reported globally last week. This is the highest number of weekly cases reported since the beginning of the pandemic [20]. According to the WHO, the largest increase in cases was seen in the Middle East (39%) followed by a 36% rise in cases in Southeast Asia [20].

## Conclusion

While it has been observed that the omicron variant is detectable on PCR testing, there is also no evidence yet to suggest that accepted COVID-19 treatment protocols and therapeutics would in future be rendered ineffective against the Omicron variant, with a possible exception of monoclonal antibodies. On November 30, 2021, Regeneron issued a statement saying that its COVID-19 monoclonal antibody treatment might be less effective against the Omicron variant as compared to previous variants of SARS-CoV-2, thereby indicating that there may be a need to update the widely beneficial monoclonal antibody drugs, if this new variant spreads widely through the global population. So long as the Omicron variant does not configure highly-mutated spike proteins resulting into “polymutant spikes,” this variant too should be responsive to the already established treatment protocols for COVID-19 infection. Additionally, the mRNA vaccines currently in use, along with their booster doses, should hopefully prove to be effective as well, in the fight against the Omicron variant. The discovery of the BA.2 lineage of the Omicron variant is a new development which may be indicative of a wider circulation of Omicron in the general population, than we now think.
